# Bioimpedance-based evaluation of relative leaf age in mango twigs using electrical impedance spectroscopy

**DOI:** 10.2478/joeb-2026-0004

**Published:** 2026-03-21

**Authors:** Ade Agung Harnawan, Khusnul Ain, Nuril Ukhrowiyah, Imam Sapuan, Ahmad Hoirul Basori

**Affiliations:** Doctoral of Mathematical and Science, Faculty of Science and Technology, Universitas Airlangga, Surabaya, Indonesia.; Physics, Faculty of Mathematical and Science, Universitas Lambung Mangkurat, Banjarmasin, Indonesia.; Medical Instrumentation System, Biosensor, Biomechatronics, and Biomaterials Research Group Universitas Airlangga, Surabaya, Indonesia.; Physics, Faculty of Science and Technology, Universitas Airlangga, Surabaya, Indonesia.; Faculty of Computing and Information Technology King Abdudaziz University, Rabigh, Saudi Arabia.

**Keywords:** Electrical impedance spectroscopy, charge transfer resistance, double-shell equivalent circuit, membrane capacitance, relative leaf age

## Abstract

This study proposes a non-destructive method for estimating the relative age of mango leaves using electrical impedance spectroscopy (EIS) and a modified double-shell equivalent circuit model. Impedance measurements were conducted on six mango varieties using five leaves per variety representing different positions along the twig. The Nyquist plots showed increasing circular arc diameters with leaf position, indicating higher charge transfer resistance in older leaves. Fitting results revealed that R1 increased while C1 decreased systematically with leaf age. Spearman correlation analysis confirmed a strong positive correlation between R1 and leaf position and a strong negative correlation for C1, whereas n1 showed no significant relationship. Linear regression yielded high coefficients of determination for most varieties. The selection of R1 and C1 as electrical indicators was supported by their low fitting errors, strong correlations, and consistent regression performance. These results demonstrate that EIS provides a rapid and reliable non-destructive approach for assessing the physiological age of mango leaves.

## Introduction

Identifying the physiological age of leaves, commonly referred to as senescence, is an important topic in agronomy, plant physiology, and ecology. Leaf senescence is an active and genetically regulated process characterized by chlorophyll degradation, loss of cellular turgor, and progressive modification of cellular structure [1]. In this context, leaf age can be defined either as chronological age, which represents the time elapsed since leaf emergence, or as relative age, which reflects the physiological and developmental status of a leaf in relation to other leaves on the same twig. The latter provides a more practical indicator of plant condition under natural growth variability and is therefore the focus of this study.

Conventional methods for determining leaf physiological age are mainly destructive or based on visual assessment, including chlorophyll content analysis, water status measurement, microscopic observation, and the leaf measuring-interval index (LMI) [2]. Although these approaches provide useful information, they are time-consuming, labor-intensive, and unsuitable for rapid or in situ monitoring. Consequently, there is a growing need for non-destructive techniques capable of assessing leaf physiological status based on intrinsic tissue properties.

Electrical impedance spectroscopy (EIS) offers a promising alternative because it characterizes the electrical response of biological tissues over a wide frequency range [3,4]. In plant tissues, impedance is strongly influenced by the structural and functional properties of cell walls, membranes, cytoplasm, and vacuoles. Within the framework of the double-shell model, these components can be represented by an equivalent electrical circuit consisting of extracellular resistance, cytoplasmic resistance, vacuolar resistance, and membrane capacitances associated with the plasmalemma and tonoplast [5]. By analyzing Nyquist plots derived from impedance measurements, these parameters can be quantitatively extracted [6]. Variations in these electrical properties, such as increases in extracellular resistance associated with cell-wall thickening, and alterations in membrane capacitances indicative of structural or functional membrane degradation, have been shown to correlate strongly with the progression of leaf senescence [7].

EIS measurements on plant tissues are commonly performed using a four-electrode configuration to minimize the influence of electrode–sample contact resistance [4,8]. However, for field applications requiring rapid measurement and minimal sample preparation, a two-electrode configuration is often preferred due to its simplicity and portability [9,10]. Although two-electrode measurements inherently include contact resistance, this limitation can be reduced when the analysis focuses on relative impedance changes or when appropriate frequency ranges and probe positioning are used [11,12]. Therefore, a two-electrode configuration remains suitable for evaluating relative leaf age.

Recent advances in portable and low-cost data acquisition systems have further expanded the applicability of EIS in biological research. The Analog Discovery 2 (AD2), originally developed as a mixed-signal oscilloscope and waveform generator, can be adapted for impedance measurements over a broad frequency range [13]. Its portability, cost efficiency, and software flexibility make it particularly attractive for plant-based measurements conducted outside controlled laboratory environments [14].

Despite extensive studies on the application of EIS in plant physiology, several challenges remain. Previous works have typically employed a single equivalent circuit model without systematically evaluating the relationship between model validity, parameter reliability, and measurement configuration. In addition, the identification of specific impedance parameters as quantitative indicators of relative leaf age has not been comprehensively addressed.

To overcome these limitations, this study applies a modified double-shell equivalent circuit based on the Hayden model proposed by Zhang and Willison [5,15]. This model enables improved separation of relaxation mechanisms and provides deeper insight into the electrical characteristics associated with leaf senescence [16]. Furthermore, a parameter-selection strategy based on fitting accuracy, correlation analysis, and regression performance is introduced to identify reliable electrical indicators of relative leaf age.

Therefore, the aim of this study is to develop a rapid and non-destructive method for estimating the relative physiological age of mango leaves using electrical impedance spectroscopy. Specifically, the objectives are to (i) evaluate the suitability of a two-electrode EIS configuration for leaf measurements, (ii) validate the modified double-shell equivalent circuit model for representing leaf impedance, and (iii) identify impedance parameters that reliably correlate with relative leaf age.

## Materials and methods

**Fig. 1:**
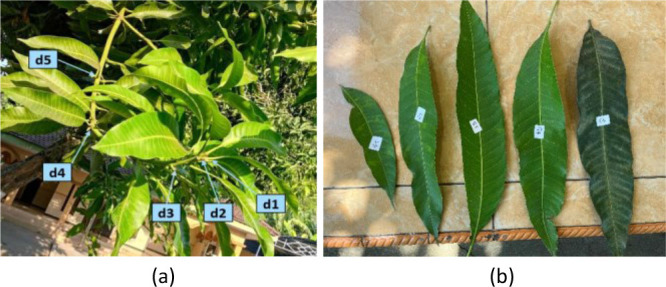
Mango leaf sample in twig (a) The position of five leaves on the twig, (b) the five samples with symbols d1, d2, d3, d4 and d5.

Leaf samples were collected from six mango varieties, namely Gadung, Manalagi, Golek, Janis, Podang, and Apel. For each variety, five leaves were taken from the same twig according to their position from the shoot tip to represent different relative leaf ages. The leaves were labeled sequentially as d1 (youngest) to d5 (oldest), as shown in Fig. 1.

**Fig. 2:**
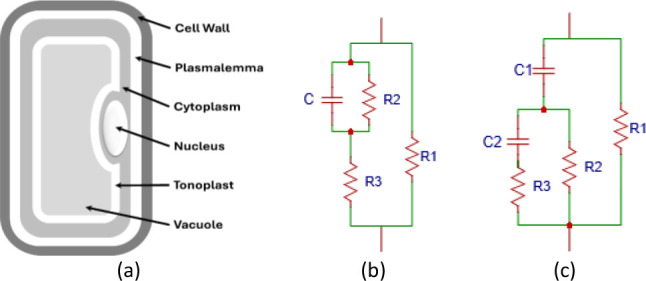
Approximation structure model. (a) The anatomical structure of a fruits and vegetables [18], (b) Hayden equivalent electrical circuit [18], (c) double-shell equivalent electrical circuit [19].

In plant cells shown in Fig. 2 (a), the extracellular cell wall surrounds each cell, while the internal boundary is defined by the phospholipid bilayer of the plasmalemma. Enclosed within the plasmalemma, the nucleus resides in the viscous cytoplasmic matrix [17]. The cytoplasm is internally delimited by the tonoplast, a specialized membrane that encloses the vacuole.

A more sophisticated model, proposed by Hayden et. al. [20] in anatomical structure, illustrated in Fig. 2 (b), characterizes the electrical properties of plant cells by assigning R1 to the cell wall resistance, R2 to the cell membrane resistance, R3 to the cytoplasmic resistance, and C to the combined capacitance of all membranes. A sub-sequent and more detailed representation—the double-shell model—that was introduced by Zhang and Willison [21] is depicted in Fig. 2 (c). In this framework, R1 denotes the cell wall resistance or extracellular resistance, R2 the cytoplasmic resistance, R3 the vacuolar resistance, C1 the plasmalemma capacitance, and C2 the tonoplast Capacitance. Later, we use the double-shell model to analyze the sample.

### Electrical impedance measurement

Leaf sample impedance measurements were conducted using the two-point method [9,13], with two probes [22] placed on the underside of the leaf blade. Each leaf was first cleaned, then two Ag/AgCl electrocardiogram (ECG) electrodes were attached to the underside (abaxial) surface of the leaf blade, 3 cm apart.

**Fig. 3:**
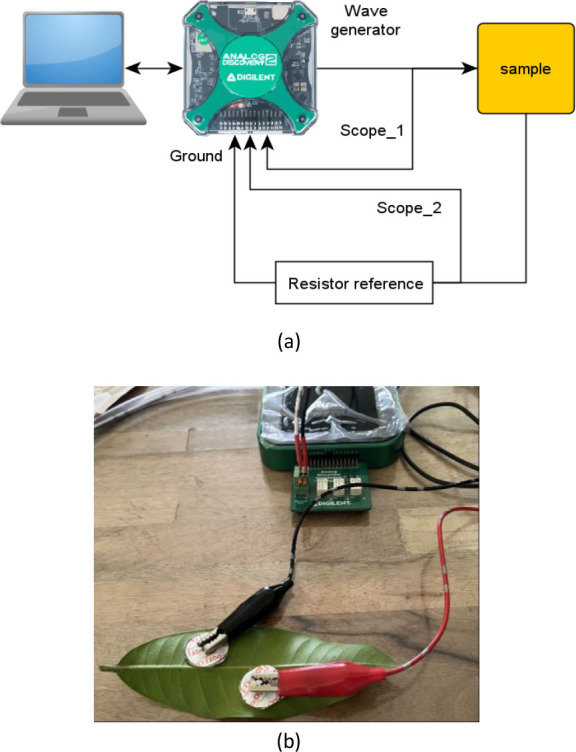
Design of sample impedance measurement. (a) the scheme measurement, (b) the impedance process measurement with the Analog Discovery 2 and the result displayed on computer.

The impedance measurements for each leaf were conducted using Electrical Impedance Spectroscopy (EIS) with the Analog Discovery, as shown in Fig. 3. Measurements were performed within a maximum of one hour of leaf collection to minimize physiological changes due to aging or water loss.

The measurements were carried out using an Analog Discovery 2 device controlled by Waveforms software [13]. A sinusoidal excitation signal of 200 mV was applied over a frequency range of 5 kHz to 100 kHz at 26 frequency points. Complex impedance data *Z* were recorded at each frequency, including the impedance magnitude ∣*Z*∣ and phase angle *θ*.

All measurements were conducted within one hour after harvesting to minimize physiological changes due to water loss. During measurement, the samples were placed on an insulating surface to avoid electrical leakage and external capacitive effects. The measurements were performed at room temperature under stable environmental conditions.

### Equivalent circuit modeling and Nyquist fitting

**Fig. 4:**
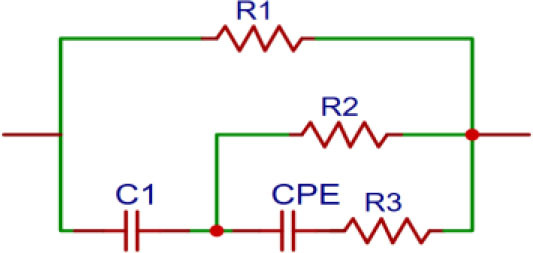
The modified double-shell equivalent electrical circuit.

The impedance data were plotted in Nyquist form and fitted using a modified double-shell equivalent circuit model. The double-shell model is built with cell wall resistance (R1), cytoplasmic resistance (R2), vacuole resistance (R3), plasma membrane capacitance (C1), and tonoplast capacitance (C2). The model can be seen in Fig. 2(c), however, to make the fitting results closer to all data on the Nyquist graph, C2 is replaced with a CPE, and the circuit model can be seen in Fig. 4. Then, the free software for non-commercial use that was used to perform the Nyquist plot fitting, was the EISSA created by Aliaksandi Bandarenka and Genady Ragoisha [23].

### Correlation of impedance with relative leaf age

From the Nyquist fitting, the parameters R1, R2, R3, C1, and the CPE elements Q1 (admittance) and n1 (phase exponent) were obtained. Their relationships with leaf position, which represents relative leaf age, were evaluated using the Spearman rank correlation test. Based on the correlation results, the most relevant parameters were identified, with R1 representing tissue resistance, C1 reflecting membrane capacitance, and n1 describing membrane heterogeneity. Parameters that showed significant correlation were then subjected to linear regression analysis to develop a model for estimating relative leaf age. The resulting regression model was subsequently applied to the other mango varieties.

### Ethical approval

The conducted research is not related to either human or animal use.

## Results

### Impedance characteristics of Mango leaves

Impedance measurements were performed on six mango varieties using five leaf samples for each variety. The Bode plots showed that the impedance magnitude decreased with increasing frequency for all samples, while the phase angle exhibited a curved pattern over the measured frequency range (5–100 kHz). Younger leaves (d1) had lower impedance and higher phase values, whereas older leaves (d5) showed higher impedance and lower phase values.

The real part of impedance (Z′, resistance) at low frequency increased progressively from d1 to d5, indicating higher tissue resistance in older leaves. The imaginary part (Z″, reactance) was negative for all samples and its magnitude changed with leaf position, reflecting variations in the capacitive behavior of the tissue.

### Nyquist plot analysis

**Fig. 5:**
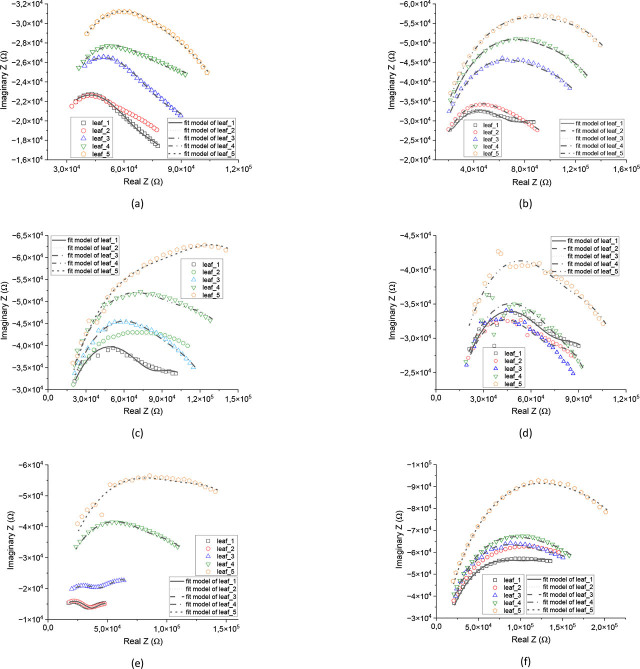
The Nyquist plot in six samples with fitting plot; (a) Gadung; (b) Manalagi; (c) Golek; (d) Janis; (e) Podang; (f) Apel.

The Nyquist plots of all mango varieties in Fig. 5 exhibit depressed circular arc patterns, indicating that the electrical response of the leaf tissue is dominated by charge transfer processes. A systematic increase in the circular arc diameter from d1 to d5 is observed for all varieties. This widening along the Z′ axis reflects an increase in charge transfer resistance (Rct) with leaf position, which represents relative leaf age. The enlargement of the circular arc suggests higher extracellular and intracellular resistance in older leaves.

In addition, the maximum negative Z″ value (the depth of the circular arc) changes with leaf position. Younger leaves show smaller Z″ magnitudes, while older leaves exhibit deeper circular arcs, indicating changes in the capacitive behavior of the cell membrane. These graphical features demonstrate a transition from a more conductive and capacitive state in young leaves to a more resistive state in older leaves.

Clear varietal differences are also observed. The Gadung variety (Fig. 5(a)) shows a gradual increase in circular arc diameter and a moderate shift toward higher Z′ values, indicating a progressive increase in tissue resistance. Manalagi (Fig. 5(b)) exhibits the most distinct separation between d1 and d5, with a pronounced horizontal expansion and higher Z″ peak, revealing a strong increase in Rct and high sensitivity to leaf aging. Golek (Fig. 5(c)) presents a uniform and stepwise enlargement of the circular arc, indicating a slow and homogeneous change in tissue electrical properties.

In contrast, Janis (Fig. 5(d)) shows small circular arcs with minimal separation between leaf positions, suggesting only minor changes in resistance and capacitive behavior and therefore the lowest sensitivity to relative leaf age. Podang (Fig. 5(e)) displays a sharp increase in circular arc diameter in older leaves, characterized by a large shift in Z′ and deeper Z″ values, indicating a substantial rise in Rct. The Apel variety (Fig. 5(f)) shows well-separated circular arcs with a consistent shift toward higher Z′ values, demonstrating systematic changes in both resistive and capacitive components.

The Nyquist plots for all varieties clearly show that increasing leaf age results in higher resistive components and modified capacitive behavior. Among the studied varieties, Manalagi and Podang exhibit the highest sensitivity to relative leaf aging, whereas Janis shows the lowest. These graphical trends confirm that EIS provides a reliable non-destructive indicator for estimating the relative age of mango leaves.

### Leaf sample relative age approach using the modified double-shell electronic model

The modified double-shell equivalent circuit shown in Fig. 4 provided a good agreement with the experimental Nyquist plots in Fig. 5 for all mango varieties, as indicated by the relatively low and consistent fitting errors for most parameters. The fitted curves closely followed the measured data, confirming that the model adequately represents the electrical behavior of mango leaf tissue.

The fitting yielded the parameters R1, R2, R3, C1, and the constant phase element (Q1 and n1) for all leaf samples (d1–d5). A systematic trend with leaf position was observed, where R1 and R2 increased with increasing leaf age in almost all varieties, while C1 decreased.

The error values demonstrate differences in the fitting quality among varieties. Gadung showed the lowest and most stable errors for R1, R2, and C1 across all leaf positions, indicating that the modified double-shell model provides the most accurate representation for this variety. Apel and Manalagi also exhibited relatively low fitting errors, confirming good model suitability.

In contrast, Podang showed a significant increase in fitting error for older leaves, particularly in the resistive components, suggesting greater structural and physiological changes that are not fully captured by the model at advanced leaf age. Larger and less consistent errors were also observed in Janis and Golek for several parameters, indicating a lower fitting accuracy compared to the other varieties.

The CPE exponent n1 ranged from 0.63 to 0.88 with relatively small fitting errors and only minor variation with leaf position, indicating a similar degree of capacitive dispersion for leaves of different ages.

The combination of low fitting errors and the close overlap between experimental and simulated Nyquist plots confirms that the modified double-shell model is suitable for describing the impedance response of mango leaves. The fitting results further show that leaf aging is mainly characterized by an increase in resistive components, while the capacitive dispersion remains relatively unchanged.

### Correlation of Impedance with Relative Leaf Age

The correlation between impedance parameters derived from Nyquist plot fitting and relative leaf age, expressed as the leaf position (3, 5, 7, 9, 11) from the stem tip, was assessed using the Spearman correlation test. The strong correlation with leaf position is the parameters C1, R1 and n1 so it is necessary to determine the correlation level using Spearman correlation and it is obtained in the Table 1.

**Table 1.** Spearman’s correlation analysis.

**No**	**Mango Variety**	**C1**		**R1**		**n1**	
		**ρ**	**p-value**	**ρ**	**p-value**	**ρ**	**p-value**
1	Manalagi	−1.0	-	0.9	0.037	0.1	0.872
2	Gadung	−0.5	0.391	1.0	-	−0.6	0.284
3	Janis	−1.0	-	0.1	0.873	0.5	0.391
4	Apel	−1.0	-	0.9	0.037	0.8	0.104
5	Podang	−1.0	-	1.0	-	0.6	0.284
6	Golek	−1.0	-	0.9	0.037	0.8	0.104

Table 1 showed a negative relationship between position and C1 across all mango varieties, with five varieties (Manalagi, Janis, Apel, Podang, and Golek) showing a perfect negative correlation (ρ = −1). This indicates that increasing position is consistently accompanied by a decrease in C1 values across almost all varieties.

The opposite pattern applies to the relationship between position and R1. Most varieties showed a strong to perfect correlation (ρ = 0.9–1), with three varieties (Manalagi, Apel, and Golek) having a statistically significant relationship (p < 0.05). This indicates that increasing position is closely related to increasing R1 values across most mango varieties

Conversely, the relationship between position and n1 showed an inconsistent pattern, both in direction and strength. All varieties had p values > 0.05, indicating that the relationship was not significant. Therefore, position did not significantly affect n1 values across all mango varieties measured.

**Fig. 6.**
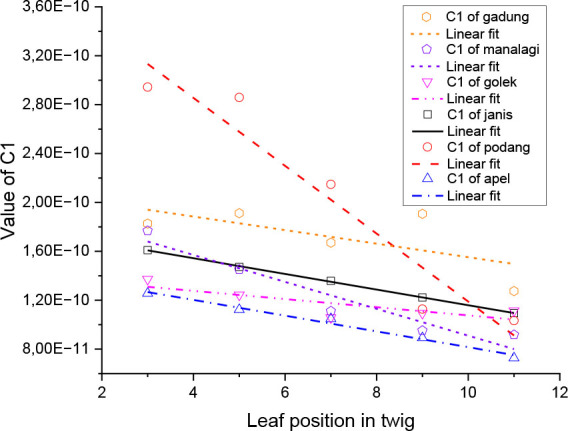
Linear regression of C1.

**Fig. 7.**
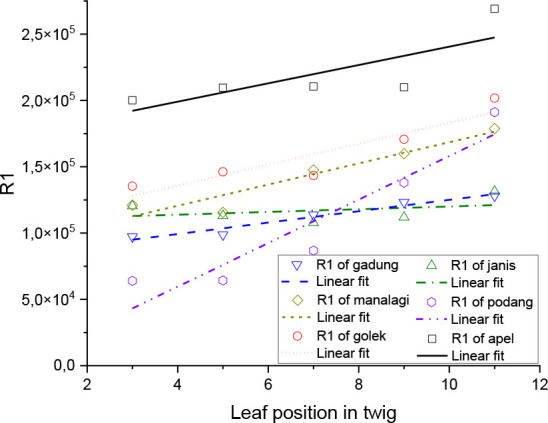
Linear regression of R1.

The depiction of the relationship between C1 and R1 values, which have a strong correlation with the sample position representing the relative age of the leaves, is shown by linear regression in Fig. 6 and 7, with the regression parameters both shown in Tables 2 and 3 respectively.

**Table 2.** Regression parameters of C1.

**Variety**	**a (intercept)**	**b (slope)**	**R^2^**
Gadung	2.11E-10 ± 2.76E-11	-5.54E-12 ± 3.66E-12	0.434
Manalagi	2.01E-10 ± 1.43E-11	-1.10E-11 ± 1.89E-12	0.918
Golek	1.41E-10 ± 1.05E-11	-3.34E-12 ± 1.39E-12	0.657
Janis	1.79E-10 ± 7.17E-13	-6.41E-12 ± 9.50E-14	0.999
Podang	3.97E-10 ± 3.52E-11	-2.78E-11 ± 4.66E-12	0.921
Apel	1.46E-10 ± 3.49E-12	-6.44E-12 ± 4.63E-13	0.984

**Table 3.** Regression parameters of R1.

**Variety**	**a (intercept)**	**b (slope)**	**R^2^**
Gadung	82064.5 ± 4311.54	4311.5 ± 571.08	0.949
Manalagi	88630.0 ± 10984.68	7790.0 ± 1454.95	0.909
Golek	104355.0 ± 14714.80	7875.0 ± 1949.02	0.844
Janis	109730.0 ± 11997.19	1030.0 ± 1589.06	0.123
Podang	−6070.5 ± 2528.24	16415.5 ± 3348.76	0.889
Apel	171435 ± 23819.12	6915.0 ± 3154.92	0.616

The graph in Fig. 6 shows a negative linear relationship between C1 and leaf position, which represents the relative leaf age, for all mango varieties, indicating that C1 decreases as the leaf becomes older. This trend reflects the reduced charge storage capacity of the leaf tissue due to the loss of membrane integrity and water content during aging. The negative slope values in all varieties shown in Table 2 confirm this decline, with the steepest decrease observed in Podang (−2.78×10^−11^) and Manalagi (−1.10×10^−11^), while Golek shows the most gradual change (−3.34×10^−12^). The highest intercept in Podang indicates the largest initial C1 value in young leaves. Based on the coefficient of determination (R^2^), C1 and relative leaf age is very strong in Janis (0.999), Apel (0.984), Podang (0.921), and Manalagi (0.918), moderate in Golek (0.657), and weak in Gadung (0.434). Overall, these results demonstrate that C1 is a sensitive capacitive parameter for assessing relative leaf age, particularly in the Janis, Apel, Podang, and Manalagi varieties.

The graph in Fig. 7 shows the relationship between R1 and leaf position, which represents the relative leaf age, for all mango varieties. In general, R1 tends to increase with increasing leaf position, indicating higher charge transfer resistance in older leaves due to reduced water content and decreased membrane integrity. This trend is confirmed in Table 3 by the positive slope values for Gadung (4300.5), Managi (7790.0), Golek (7875.0), Podang (16415.5), and Apel (6915.0), with Podang showing the steepest increase and thus the highest sensitivity to relative leaf age, and Janis (1030.0) exhibited the smallest slope, indicating only a minor change across leaf positions. The intercept values indicate the initial R1 of young leaves, with Apel having the highest starting value. Based on the coefficient of determination (R^2^), the linear relationship is very strong in Gadung (0.949), Manalagi (0.909), and Podang (0.889), good in Golek (0.844), moderate in Apel (0.616), and very weak in Janis (0.123). Overall, these results show that R1 is a reliable resistive parameter for estimating relative leaf age, particularly in Gadung, Manalagi, Podang, and Golek varieties, while its sensitivity is limited in Janis.

## Discussion

The systematic change in impedance parameters with leaf position demonstrates that the electrical response of mango leaves is strongly influenced by physiological aging. The frequency-dependent decrease in impedance confirms the dispersive behavior of biological tissues, where membrane polarization dominates at low frequencies and current penetration into intracellular regions increases at higher frequencies [24,25].

The increase in Z′ at low frequencies and the enlargement of the Nyquist circular arc indicate an increase in charge transfer resistance with leaf age. This reflects reduced ionic conductivity in both extracellular and intracellular pathways, which is commonly associated with water loss, increased cell wall rigidity, and reduced electrolyte mobility during leaf maturation [26].

The Nyquist plots exhibited circular patterns for all varieties, indicating that the electrical response was dominated by charge transfer processes. The progressive enlargement of the circular arc with leaf age demonstrates an increase in charge transfer resistance. This behavior can be attributed to structural changes in leaf tissue, including decreased membrane permeability, reduced intracellular conductivity, and increased mechanical rigidity of the cell wall. Such changes limit ion transport and are characteristic of aging plant tissues [27].

The modified double-shell model provided a good representation of the impedance spectra, confirming that the electrical behavior of mango leaves can be described in terms of extracellular resistance (R1), cytoplasmic resistance (R2), and membrane capacitance (C1). The increase in R1 and R2 with leaf age indicates that both extracellular and intracellular pathways become more resistive. This is consistent with the reduction in water content and the reorganization of cellular structures during leaf development and senescence [20].

The decrease in C1 with leaf position indicates a reduction in membrane capacitance. Since membrane capacitance is proportional to membrane integrity and effective surface area, this trend suggests progressive structural degradation of the plasma membrane. Similar behavior has been reported in aging plant tissues, where lipid peroxidation and membrane reorganization reduce charge storage capability [28].

The relatively constant n1 values indicate that the degree of capacitive dispersion remained similar across leaf ages. Therefore, the dominant electrical changes during aging were associated with the resistive components rather than changes in membrane heterogeneity, which is consistent with previous bioimpedance studies on plant tissues [27].

The correlation analysis demonstrated that R1 and C1 are the most sensitive parameters for describing relative leaf age. The strong positive correlation between R1 and leaf position indicates that tissue resistance increases as leaves mature, whereas the strong negative correlation between C1 and leaf position reflects the loss of membrane capacitance. The absence of a significant correlation for n1 confirms that membrane heterogeneity is not the primary factor controlling the aging response.

The regression models further show that both parameters can be used to estimate relative leaf age quantitatively. The higher sensitivity observed in Manalagi and Podang indicates that these varieties undergo more pronounced structural and physiological changes during leaf development, whereas the weak response in Janis suggests more stable tissue properties.

Overall, the combined decrease in C1 and increase in R1 demonstrate a transition from a capacitive and conductive state in young leaves to a more resistive state in older leaves. This electrical transition reflects the underlying biophysical processes of leaf aging, including water redistribution, membrane degradation, and cell wall modification. These findings confirm that electrical impedance spectroscopy provides a reliable, non-destructive approach for assessing the relative physiological relative age of mango leaves.

## Conclusion

This study demonstrates that electrical impedance spectroscopy combined with a modified double-shell equivalent circuit model enables non-destructive estimation of the relative physiological age of mango leaves. The two-electrode configuration provided stable and reliable measurements, and the model showed good agreement with the experimental spectra, allowing extraction of physically meaningful parameters. The increase in R1 and the decrease in C1 with leaf position indicate a transition from a conductive–capacitive state in young leaves to a more resistive state in older leaves. These parameters exhibited the strongest correlation and regression performance and were identified as robust electrical indicators of leaf aging. This work establishes a systematic parameter-selection framework based on model validity and statistical analysis and highlights the potential of impedance-based techniques for rapid, non-destructive assessment of plant physiological status.
